# Intraoperative Transient Central Diabetes Insipidus Status Post-Cerebellopontine Meningioma Resection: A Case Report

**DOI:** 10.7759/cureus.66382

**Published:** 2024-08-07

**Authors:** Bright O Etumuse, Stephen Arhewoh, Amit Aggarwal, Urmil Patel, Darsh S Shah, Pablo Valdez Quevedo, Daniel Arango

**Affiliations:** 1 Medical Education, University of Texas Medical Branch at Galveston, Galveston, USA; 2 Anesthesiology, University of Texas Medical Branch at Galveston, Galveston, USA; 3 Neurosurgery, University of Texas Medical Branch at Galveston, Galveston, USA; 4 Neurobiological Surgery, University of Texas Medical Branch at Galveston, Galveston, USA

**Keywords:** intraoperative fluid management, intraoperative neurologic monitoring, intraoperative monitoring, meningioma, cerebellopontine angle, neurosurgery, vasopressin, desmopressin, diabetes insipidus, anesthesiology

## Abstract

Central diabetes insipidus (CDI) is a neurological pathological condition in which vasopressin synthesis has been compromised. A 52-year-old male presented with a cerebellopontine angle mass not involving the hypothalamic-pituitary axis. Despite vasopressin therapy, the patient produced a total of 8650 mL of urine, with the urine-specific gravity measured at 1.002 near hour 8. A literature review found associations with certain anesthetic drugs that have an increased incidence of CDI, including alpha-2 agonists and sevoflurane. Reports have recommended administering desmopressin over vasopressin, especially for neurosurgery cases that warrant a more extended operative period, given that desmopressin has a longer context-sensitive half-life.

## Introduction

Central diabetes insipidus (CDI), also known as neurogenic diabetes insipidus, is a pathological condition resulting from compromised vasopressin synthesis, leading to symptoms such as polydipsia and polyuria [1]. Vasopressin, also called arginine vasopressin (AVP), is synthesized by magnocellular neurosecretory neurons in the paraventricular nucleus (PVN) and the supraoptic nucleus (SON) of the hypothalamus [1]. AVP exerts anti-diuretic effects on the kidneys via vasopressin 2 (V2) receptors on principal cells in the collecting duct and vasoconstriction via vasopressin 1 (V1) receptors in vascular smooth muscle cells [2]. Although about 25% of CDI cases are idiopathic, many cases occur after neurohypophyseal damage, and clinical symptom severity correlates to the degree of damage to the neurohypophysis.

Traumatic events or surgeries near the neurohypophysis can lead to both intraoperative and postoperative CDI. In some cases, a transient CDI state occurs immediately postop and typically resolves within weeks. However, some reports have shown refractory postoperative CDI, where there is over 85% loss of the hypothalamic magnocellular neurons [3]. The initial phase occurs due to axon shock, with a subsequent second phase involving an unregulated release of synthesized AVP, which manifests with high anti-diuretic effects, lasting a few days to two weeks [4]. The third phase occurs after complete AVP release and presents with symptoms of CDI [5,6]. Further research is indicated for intraoperative diagnosis and intervention of clinically suspected CDI.

The first step in diagnosing intraoperative CDI is to establish the presence of hypotonic polyuria. Patients with urine output greater than 2 mL/kg/hour and a persistent urine volume greater than 200 mL to 300 mL/hour should raise suspicion for CDI [7]. Laboratory tests that may support the diagnosis may include serum hyperosmolality and rapid onset hypernatremia [8]. Here, we introduce a case involving a craniotomy for the resection of the cerebellopontine angle (CPA) meningioma complicated by intraoperative CDI. Written Health Insurance Portability and Accountability Act (HIPAA) authorization was obtained from the patient for this case report's publication.

The case report aims to expound upon a case of intraoperative CDI with unknown etiology during a CPA mass excision under a craniotomy in a 59-year-old male with a history of right frontal lobe meningioma.

This article was previously presented as a meeting abstract at the 2023 International Anesthesia Research Society (IARS)/Society of Critical Care Anesthesia Annual Conference (SOCCA) on April 13, 2023.

## Case presentation

**Figure 1:**
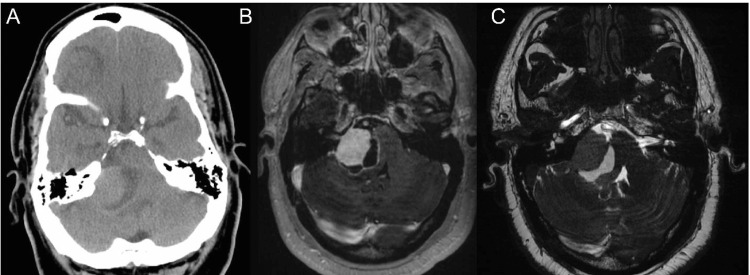
Preoperative images Axial (A) non-contrast CT, (B) contrast-enhanced T1-weighted MRI, and (C) high-resolution 3D FIESTA images Images demonstrate a ~3.5 cm contrast-enhancing mass in the right CPA exerting a mass effect on and displacing the brainstem with effacement of the fourth ventricle. FIESTA: fast imaging employing steady-state acquisition

A 52-year-old male with diabetes mellitus, essential hypertension, and a past surgical history of a right frontal lobe meningioma World Health Organization (2021 WHO) grade 1 status post-resection in 2008 presented to his primary care physician for evaluation of a two-week history of worsening occipital headache and difficulty speaking. Prior to admission, he did not complain of significant polyuria or polydipsia. The physical exam was notable for decreased hearing on the right, a right facial weakness House-Brackmann 2, and abnormal gait. Routine laboratory tests were unremarkable. CT head without contrast showed a 3.5 cm solid-cystic mass in the right CPA with a mass effect on the brainstem. Next, MRI showed a mixed solid-cystic contrasting-enhancing mass extending along the petrous face in the CPA with possible extension into the internal acoustic canal (IAC), jugular foramen, and hypoglossal canal (Figure 1). The patient was admitted for resection of the right CPA mass in a joint case with neurosurgery and neurotology.

Anesthesia pre-procedure evaluation showed an American Society of Anesthesiologists (ASA) of physical status classification system class 3. His renal evaluation was unremarkable, with normal urine output, no electrolyte abnormalities, and a normal creatinine value. The patient was cleared by the anesthesia team for surgery.

Intraoperative course

Induction of total intravenous anesthesia with propofol and sufentanil was unremarkable. The patient was placed supine with the head turned left for a planned right retro-sigmoid craniotomy. A linear incision was made two fingers posterior to the pinna, extending 2 cm above and below the pinna. A wide craniotomy with a partial mastoidectomy was performed. The craniotomy included skeletonization of the sigmoid and transverse sinuses to the level of the sigmoid as it turned anteriorly and extended inferiorly down to 1.5 cm from the foramen magnum and 4 cm posteriorly from the level of the sigmoid sinus to ensure optimal access to the large CPA mass. The mass was resected using the standard microsurgical technique, with concurrent cranial nerve neuromonitoring to ensure the preservation of cranial nerve function.

Tumor debulking continued until cranial nerves VII-XII were identified with positive stimulation down to 0.05 mA at the end of surgery. A residual tumor was left due to meningioma invasion of the IAC, jugular foramen, and hypoglossal canal with adherence to cranial nerves VII-XII. The tumor was highly adherent to the brainstem, which led to a small thin layer of residual tumor adherent to the brainstem. An autologous abdominal fat graft was utilized, and the IAC was meticulously sealed to minimize cerebrospinal fluid (CSF) leak risks. Dural closure incorporated a suturable dural graft and craniectomy reconstruction aimed to diminish potential CSF leak risks. The surgical site underwent meticulous layer-wise closure in standard fashion.

**Figure 2:**
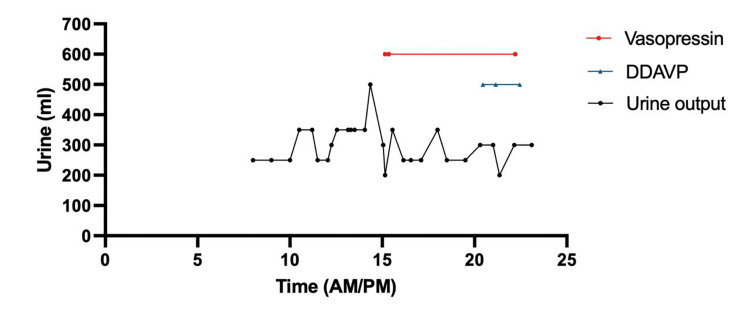
Intraoperative urine output (mL) with simultaneous vasopressin (16.68 units) and desmopressin (12 mcg) administration

**Figure 3:**
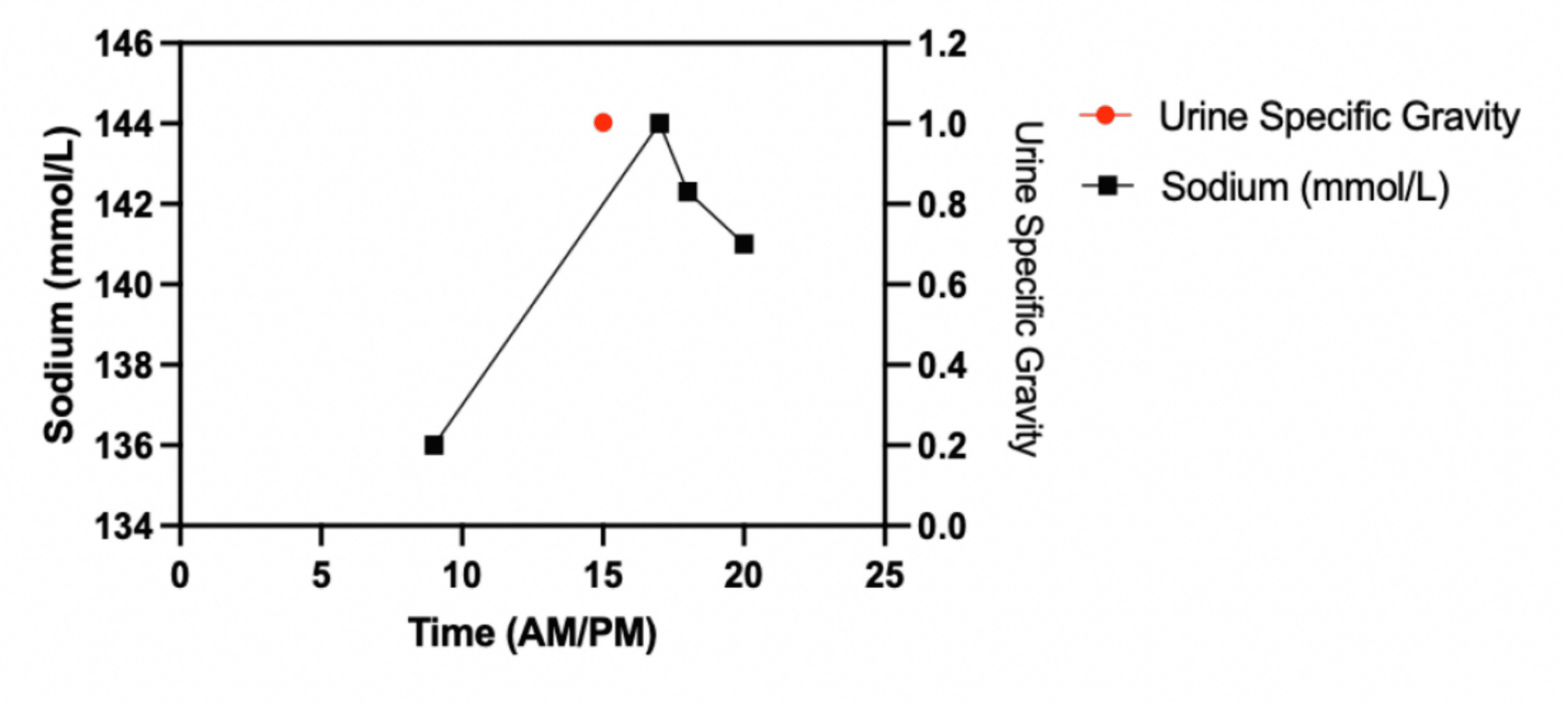
Intraoperative sodium monitoring with urine specific gravity (1.002 @ 1500)

Within the first three hours of surgery, he had produced ~750 mL of urine. This increased to ~5000 mL of urine produced at hour 8. Discussions commenced between the neurosurgery and anesthesia teams, and the patient was then started on 0.02 - 0.04 units/min of vasopressin. The patient continued to have increased urine output despite vasopressin therapy. At hour 13, the patient was started on 4 mcg of intravenous desmopressin (DDAVP). The patient produced another ~1300 mL over the following two hours, necessitating an additional 8 mcg DDAVP. During this time, plasma sodium continued to rise, and the urine-specific gravity was under expected limits. The surgery ended at approximately 12:40 AM with a total amount of 8650 mL of urine produced intraoperatively (Figures 2-3).

Postoperative course

Post-resolution of the patient's transient CDI, he was admitted to the neurological intensive care unit (ICU) post-op while intubated. 0.02 - 0.04 units/min infusion of Vasopressin and Desmopressin (12 mcg total) were discontinued after the procedure. Following surgery, the patient remained in the ICU for about one month before being discharged. His post-surgical ICU stay was mainly unrelated to the transient DI experienced during his surgery. A significant problem encountered during his ICU stay was failure to extubate, complicated by excessive oral secretions and respiratory failure, requiring multiple re-intubations and subsequent tracheostomy tube placement. Additional problems encountered included AKI requiring CRRT and suspected GI bleeding, both of which were ultimately resolved before discharge. The patient gradually improved post-discharge from the hospital and was followed by neurosurgery and otolaryngology for one year.

## Discussion

CDI was first defined in 1948 by hypernatremia (serum sodium concentration >145 mmol/l) or excretion of large volumes of dilute urine (osmolality <250 mmol/kg) [9]. The pharmacological treatments for CDI include vasopressin and analogs of vasopressin such as DDAVP. DDAVP acts on the same vasopressin receptors but has a longer context-sensitive half-life due to the removal of the amine group from vasopressin. Vasopressin has a short duration of action of about two to eight hours, contains vasoconstrictor and uterotonic properties, and is thus primarily utilized in acute CDI [9].

CDI can also be secondary to autoimmune conditions. A newfound potential cause of CDI involves immunoglobulin G4 (IgG4)-producing cells and is subsequently named IgG4-related infundibulo-hypophysitis (IgG4-RH) [10]. Lymphocytic hypophysitis is an autoimmune disorder characterized by T and B lymphocytes infiltrating the pituitary gland, with subsequent inflammation and dysfunction leading to clinical manifestations such as CDI [11]. IgG4-RH and lymphocytic hypophysitis are identical histologically, but elevated serum IgG4 levels and other systemic diseases mediated by IgG4 antibodies distinguish both pathologies [12]. Rarely, CDI may be caused by infectious etiologies such as tuberculosis and toxoplasmosis [13]. Previous case reports have described clinical courses involving tuberculomas or tuberculous meningoencephalitis extending to the hypophysis and leading to CDI [14,15].

Despite similar presentations, detecting CDI secondary to surgical events is distinct from other etiologies. Physicians typically rule out other potential causes of polyuria postop, which may include high peri-operative fluid administration, hyperglycemia, or the use of diuretic medications. The volume of fluid intake and urine output is critical for making the correct diagnosis [5]. A consensus for adult patients is to suspect CDI intraoperatively if urine output exceeds 250 mL/hour with a urine osmolarity <300 mOsm/kg or specific gravity <1.005 [6]. For children, the current literature states concern for intraoperative CDI if the urine rate is 100 ml/kg/24 hours [6]. Post-operative CDI management is critical, as hypernatremia can lead to multiple complications, including blood vessel rupture, coma, hypotension, headaches, and kidney damage [7]. The major risk of over-administering desmopressin or vasopressin is significant hyponatremia, which can lead to severe neurological complications.

Anesthetic medication management must be considered before induction in cases that run a moderate CDI risk. A recent literature review from 2022 found associations between certain anesthetic drugs and CDI incidence. Studies show that alpha-2 agonists, such as dexmedetomidine, decrease the AVP release and the nephrogenic response to AVP at continuous infusions or with a large single-loading dose of 1 ug/kg. Sevoflurane has also been found in meta-analyses to rarely cause transient CDI by impairing the aquaporin-2 response to AVP. Ketamine can inhibit glutamate, an excitatory neurotransmitter involved in the stimulation and secretion of AVP. Propofol was found to be the rarest of anesthetic drugs that can cause CDI, theoretically by the gamma-aminobutyric acid-mediated inhibition to inhibit AVP release. Human studies have not been thoroughly conducted to test these compounds; however, caution is still advised for the intraoperative inclusion of certain medications, especially with a strong suspicion of possible CDI [15].

## Conclusions

The presentation of transient intraoperative CDI for a CPA mass resection under a craniotomy was unique, with its unknown etiology. Because intraoperative CDI is rare, reports have recommended administering desmopressin over vasopressin, especially for neurosurgery cases that warrant extended operative periods. Anesthetic management for central CDI is also controlled by limiting potential triggering medications.
